# Phytochemistry, Pharmacological Potential and Industrial Applications of *Ricinus communis* L.: A Review

**DOI:** 10.3390/ph19081258

**Published:** 2026-08-10

**Authors:** Sinovuyo Mtendwa, Pamela Rungqu, Vuyani Maqanda

**Affiliations:** Department of Earth and Chemical Sciences, Chemistry Discipline, University of Fort Hare, Alice 5700, Eastern Cape, South Africa; prungqu@ufh.ac.za (P.R.); vmaqanda@ufh.ac.za (V.M.)

**Keywords:** *Ricinus communis*, medicinal plants, traditional uses, pharmacological activities, phytochemistry, industrial applications, cultivars, castor oil, toxicity

## Abstract

This review consolidates current knowledge on the phytochemical composition, traditional uses, pharmacological properties, and industrial application of *Ricinus communis* L. This plant belongs to the Euphorbiaceae family and is a globally distributed plant of considerable medicinal and industrial importance. It is rich in bioactive compounds, notably ricinoleic acid as the dominant fatty acid in seed oil, as well as ricin, ricinine, phenolic acids and flavonoids distributed across different plant parts. Variations in phytochemical profiles among cultivars and tissues are influenced by genetic and environmental influences. Traditional medicinal uses of the leaves, roots, seeds, and oil, particularly for inflammatory conditions, pain, infections, wound healing, and gastrointestinal disorders, are critically examined in relation to experimental pharmacological evidence. Castor oil extracted from the *R. communis* plant remains central to the plant’s industrial value, serving as a renewable feedstock for pharmaceuticals, cosmetics, polymers, lubricants, and biofuels due to the unique hydroxyl functionality of ricinoleic acid. However, the presence of the highly toxic protein ricin in unprocessed seeds necessitates strict processing and safety controls. Overall, *R. communis* emerges as a chemically versatile species with significant therapeutic and industrial potential, warranting further research into cultivar-specific chemistry, standardization of extraction and testing methods, and safe value-adding applications.

## 1. Introduction

Medicinal plants continue to play a fundamental role in healthcare systems worldwide and remain an important source of bioactive compounds for pharmaceutical development. Approximately 80% of the global population relies partially on plant-derived remedies for primary healthcare, while numerous modern drugs have originated from natural products or their derivatives [1]. The growing demand for sustainable therapeutics, coupled with increasing concerns regarding antimicrobial resistance, chronic inflammatory disorders, metabolic diseases, and cancer, has renewed scientific interest in medicinal plants as reservoirs of structurally diverse bioactive molecules [2,3]. Recent advances in phytochemistry, metabolomics, molecular pharmacology, and nanotechnology have further accelerated the exploration of plant-derived compounds for both therapeutic and industrial applications [4].

*Ricinus communis* L. (Euphorbiaceae), commonly known as castor bean or castor oil plant, is a multipurpose species recognized for its medicinal, industrial, agricultural, and biotechnological importance. The plant is widely distributed throughout tropical and subtropical regions and is cultivated extensively for castor oil production [5]. The exceptional industrial value of *R. communis* is largely attributed to ricinoleic acid, which constitutes approximately 85–90% of the fatty acid composition of castor oil. This unique hydroxylated fatty acid serves as an important renewable feedstock for the manufacture of pharmaceuticals, cosmetics, lubricants, polymers, surfactants, and biodiesel [6]. In addition to its industrial significance, different parts of the plant, including the leaves, roots, seeds, and oil, have long been used in traditional medicine for the treatment of pain, inflammation, infections, gastrointestinal disorders, and skin diseases [7].

Phytochemical investigations have revealed that *R. communis* contains a diverse array of biologically active compounds, including phenolic acids, flavonoids, alkaloids, terpenoids, fatty acids, and proteins. Several experimental studies have demonstrated antioxidant, anti-inflammatory, antimicrobial, antidiabetic, hepatoprotective, wound-healing, analgesic, and anticancer activities associated with these constituents. Recent studies have also highlighted the potential of castor-derived biomaterials, nanoformulations, and functional polymers for advanced biomedical and industrial applications [8]. Furthermore, molecular investigations have begun to elucidate signaling pathways involved in the biological activities of *R. communis*, including modulation of nuclear factor kappa B (NF-κB), mitogen-activated protein kinase (MAPK), cyclooxygenase-2 (COX-2), phosphoinositide 3-kinase/protein kinase B (PI3K/Akt), and nuclear factor erythroid 2-related factor 2 (Nrf2) pathways [9].

Despite these promising developments, several critical challenges continue to hinder the translation of *R. communis* into evidence-based therapeutic applications. Most pharmacological studies remain limited to in vitro assays and animal models, while robust human clinical evidence remains scarce. Considerable variability in phytochemical composition has been reported across cultivars, geographical regions, environmental conditions, and extraction methods, making standardization difficult. Furthermore, the presence of ricin, one of the most potent plant-derived toxins known, raises important toxicological and regulatory concerns that must be carefully addressed before broader medicinal applications can be recommended. Additional limitations include insufficient pharmacokinetic data, poor understanding of bioavailability, lack of standardized formulations, and limited information regarding long-term safety [10].

Although several reviews have examined specific aspects of *R. communis*, including its ethnomedicinal uses, phytochemistry, pharmacology, or industrial applications, these studies often focus on individual disciplines and provide limited integration of therapeutic potential, clinical evidence, toxicity considerations, and industrial feasibility. Furthermore, many previous reviews have largely summarized available findings without critically evaluating evidence quality, translational limitations, mechanistic insights, safety concerns, or future commercialization prospects. Consequently, a comprehensive and critical synthesis of current knowledge remains necessary.

The present review aims to provide an updated and integrated evaluation of the phytochemistry, ethnomedicinal uses, pharmacological activities, clinical evidence, toxicity profile, industrial applications, and phytoremediation potential of *R. communis*. Emphasis is placed on cultivar-dependent phytochemical variability, molecular mechanisms of action, evidence quality, clinical translational challenges, safety considerations associated with ricin exposure, and emerging opportunities in nanotechnology, omics-guided phytochemical discovery, and sustainable industrial development. By identifying current knowledge gaps and future research priorities, this review seeks to provide a framework for the responsible utilization and commercialization of *R. communis* in pharmaceutical and industrial sectors.

## 2. Botanical Description and Geographic Distribution of *R. communis* L.

*Ricinus communis* L. is a fast-growing shrub or small tree belonging to the Euphorbiaceae family. Although the species displays considerable morphological variation, its greatest scientific importance lies in its exceptional ability to produce oil-rich seeds that serve as the principal source of commercial castor oil [11]. The seeds constitute the most economically valuable part of the plant because they contain high concentrations of ricinoleic acid, the characteristic hydroxylated fatty acid responsible for the unique physicochemical properties of castor oil, while simultaneously containing ricin, one of the most potent naturally occurring plant toxins [12]. This combination of industrial value and toxicological significance distinguishes *R. communis* from most other medicinal plants.

The species is highly adaptable to diverse ecological conditions and thrives in warm tropical and subtropical environments with well-drained soils, allowing widespread cultivation for industrial oil production [13]. Female flowers develop into spiny capsules that enclose the seeds, which are harvested for pharmaceutical, cosmetic, chemical, polymer, lubricant, and biodiesel industries following appropriate processing to eliminate toxic contaminants [14,15].

*R. communis* is believed to have originated in tropical Africa, although centuries of cultivation have resulted in extensive naturalization throughout tropical and subtropical regions worldwide, making its precise center of origin difficult to define [16]. Commercial cultivation is concentrated in countries including India, Brazil, China, Argentina, Mexico, Mozambique, and the United States, where favorable climatic conditions support large-scale castor production [17]. In South Africa, the species occurs as both a cultivated and naturalized plant, particularly in Limpopo, Eastern Cape, Mpumalanga, and KwaZulu-Natal provinces [18,19]. The broad geographical distribution of *R. communis* has important implications beyond cultivation. Environmental conditions such as climate, soil characteristics, altitude, water availability, and agricultural practices influence the accumulation of ricinoleic acid, phenolic compounds, flavonoids, alkaloids, and toxic proteins, contributing to considerable geographical variation in phytochemical composition and biological activity [20,21]. These differences partly explain the variability observed among pharmacological investigations conducted in different regions and emphasize the need for cultivar-specific phytochemical standardization before therapeutic or industrial applications can be reliably compared.

## 3. Cultivar Diversity of *R. communis* L.

*R. communis* is classified as a monotypic species, indicating that the *Ricinus* genus comprises only a single species [22]. Across its many cultivars like Impala, Carmencita, Gibsonii, Red Spire, New Zealand Purple, and Zanzibarensis, the horticultural differences (height, leaf color, stem pigmentation) reflect ornamental selection [23]. These cultivars often thrive under similar favorable growth conditions: warm temperatures (ideally 20–25 °C), full sun, fertile and well-drained soils, and regular moisture during establishment, with protection from frost in colder regions [24]. For instance, dwarf forms like Impala or New Zealand Purple are well suited to sunny beds or large containers, while tall, architectural cultivars such as Red Spire or Zanzibarensis lend themselves to spacious subtropical landscapes [25,26]. Beyond morphological differences, cultivar selection significantly influences the phytochemical and industrial value of *R. communis* [27]. Studies have demonstrated substantial variation in seed oil content, ricinoleic acid concentration, ricin accumulation, and antioxidant constituents among cultivars. Such variability directly affects biodiesel quality, pharmaceutical applications, and toxicity profiles. Consequently, cultivar characterization should extend beyond agronomic traits to include metabolomic and toxicological assessments.

From a phytochemical perspective, cultivar-dependent variation significantly affects the quantity and quality of castor oil. Seed oil content generally ranges from 35 to 60%, depending on genotype and environmental conditions, while ricinoleic acid remains the predominant fatty acid, accounting for approximately 85–90% of total fatty acids [28]. However, variations in fatty acid profiles among cultivars have been reported, which may influence the physicochemical properties of castor oil and its suitability for pharmaceutical formulations, lubricants, polymers, and biodiesel production. Consequently, cultivar selection plays a critical role in determining the economic and industrial value of *R. communis* [29].

Environmental factors further contribute to phytochemical variability among cultivars. Climatic conditions, soil characteristics, water availability, and agronomic practices can alter the accumulation of secondary metabolites, phenolic compounds, flavonoids, and fatty acids, resulting in significant differences in biological activity and industrial performance. Such variability may partly explain inconsistencies observed among pharmacological studies conducted using plant materials collected from different geographical regions and underscores the need for standardized cultivation and extraction protocols [30].

Cultivar variability also has important toxicological implications. The concentrations of ricin, ricinine, and other biologically active compounds may differ among genotypes, influencing both therapeutic potential and safety profiles. Ricin remains the principal toxic constituent of *R. communis*, and its presence poses major challenges for the medicinal and industrial utilization of castor-derived products [31]. Consequently, recent breeding programs have focused not only on improving seed yield and oil productivity but also on reducing ricin content and enhancing safety characteristics. The development of low-toxin cultivars could significantly improve occupational safety during cultivation, harvesting, and industrial processing while expanding opportunities for pharmaceutical and nutraceutical applications [32].

Recent advances in genomics, metabolomics, and plant breeding have created new opportunities for the identification of elite castor cultivars with desirable chemical and agronomic traits [33]. Modern breeding strategies increasingly integrate molecular markers, metabolomic profiling, and quantitative trait analysis to optimize ricinoleic acid production, enhance stress tolerance, improve disease resistance, and reduce toxic constituents [34]. Therefore, future research should focus on comprehensive cultivar-level phytochemical characterization and metabolomic fingerprinting to facilitate standardization, improve reproducibility of pharmacological studies, and support the sustainable industrial exploitation of *R. communis*.

Table 1 summarizes the major phytochemical biosynthesis pathways identified in *R. communis*, highlighting the relationship between metabolic routes, associated genes and enzymes, and the key bioactive compounds produced. It compares lipid-derived pathways responsible for castor oil production with secondary metabolite pathways involved in phenolamide, flavonoid, and alkaloid biosynthesis. Together, these pathways explain the chemical diversity observed in the species and provide insight into both its industrial value (e.g., ricinoleic acid-rich triacylglycerols) and its biological properties, including antioxidant activity and toxicity. This framework also links recent omics-based findings to metabolite accumulation patterns across different tissues of *R. communis*.

## 4. Phytochemical Profile of *R. communis* L.

**Figure 1:**
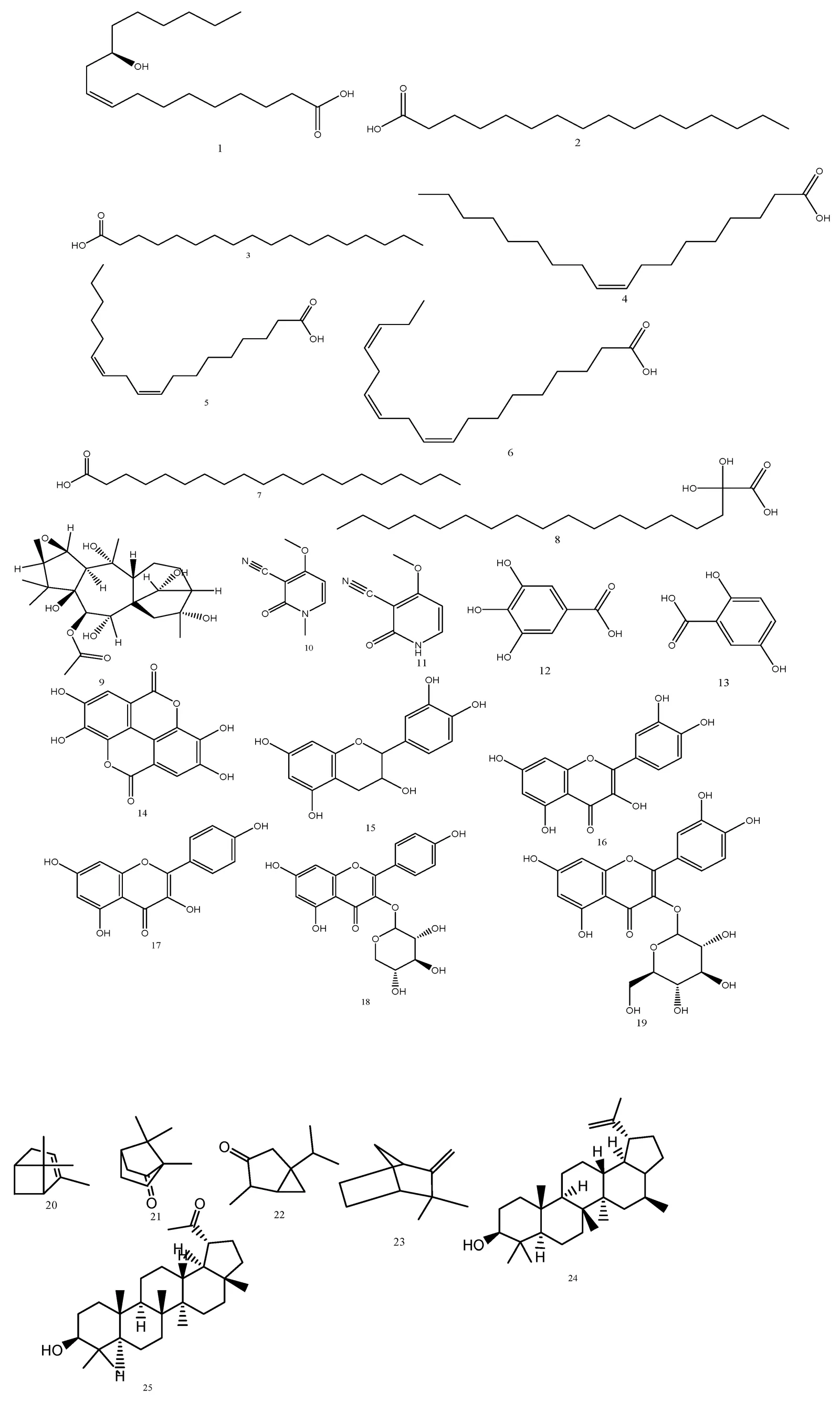
Chemical compounds in different parts of *R. communis*.

The therapeutic and industrial importance of *R. communis* is largely determined by its diverse phytochemical composition. Unlike many medicinal plants in which pharmacological activity is associated with a single class of compounds, *R. communis* contains a complex mixture of fatty acids, proteins, alkaloids, flavonoids, phenolic acids, terpenoids, sterols, glycosides, and tannins that are unevenly distributed among different plant organs [41]. The seed represents the most extensively investigated plant organ because it contains approximately 45% fixed oil, of which ricinoleic acid (1) (Figure 1) constitutes approximately 85–90% of the total fatty acid composition. Other fatty acids, including palmitic acid (2), stearic acid (3), oleic acid (4), linoleic acid (5), linolenic acid (6), arachidic acid (7), and dihydroxystearic acid (8) (Figure 1), occur in lower concentrations and contribute to the physicochemical properties of castor oil that underpin its pharmaceutical and industrial applications. Although ricinoleic acid remains relatively conserved across cultivars, measurable quantitative variation has been reported according to genotype, environmental conditions, and geographical origin, with corresponding differences in oil quality, viscosity, oxidative stability, and industrial performance [42,43,44]. *R. communis* also contains several toxic proteins and nitrogen-containing compounds, particularly in the seeds. The most significant of these is ricin (9), ricinine (10), N-demethylricinine (11) (Figure 1), a highly potent type-2 ribosome-inactivating protein (RIP2). Ricin is composed of two polypeptide chains (A and B chains) and functions by inhibiting protein synthesis in cells, making it one of the most toxic plant-derived substances. Other related proteins include *R. communis* agglutinin (RCA), which is structurally similar to ricin but less toxic [45].

Leaves represent the principal source of phenolic compounds and flavonoids. Major phenolic acids include gallic acid (12), gentisic acid (13), ellagic acid (14), and epicatechin (15), while quercetin (16), kaempferol (17), kaempferol-3-xyloside (18), and quercetin-3-O-glucoside (19) constitute the predominant flavonoids identified to date. These compounds have consistently been associated with some biological activities owing to their ability to scavenge reactive oxygen species, modulate inflammatory mediators, and regulate intracellular signaling pathways. However, quantitative concentrations differ markedly between studies, reflecting differences in cultivar, geographical origin, harvesting season, plant maturity, extraction solvent, and analytical methodology [46,47].

Volatile constituents occur mainly in essential oils obtained from leaves, stems, and seeds. Gas chromatography-mass spectrometry (GC-MS) analyses have identified α-pinene (20), camphor (21), thujone (22), camphene (23), and numerous minor terpenoids that contribute to the characteristic aroma and reported antimicrobial activity of the plant [48,49]. Terpenoids and essential oil components are present in different parts of the plant, including stems, leaves and seeds. Studies using gas chromatography–mass spectrometry (GC-MS) have identified compounds such as α-pinene (20), camphor (21), thujone (22), and camphene (23) (Figure 1) in the essential oils extracted from *R. communis* [50,51]. Other compounds such as lupeol (24) and 30-norlupan-3β-ol-20-one (25) have also been isolated from the outer layers of the seeds [52].

Available evidence demonstrates that the phytochemistry of *R. communis* is highly dynamic and influenced by both genetic and environmental factors. Future investigations should prioritize metabolomic fingerprinting, quantitative phytochemical profiling, and chemotype classification to establish robust relationships between chemical composition and biological activity. Such approaches would substantially improve reproducibility, facilitate standardization of medicinal preparations, and strengthen the translation of preclinical findings into pharmaceutical and industrial applications.

### Extraction Methods and Their Influence on Phytochemical Recovery

Extraction methodology is one of the principal determinants of phytochemical composition and, consequently, biological activity in *R. communis*. The wide variation in extraction procedures employed across published studies has contributed substantially to the inconsistent pharmacological findings reported in the literature. Differences in solvent polarity, extraction temperature, extraction time, plant part, particle size, and analytical techniques influence both the qualitative and quantitative composition of the resulting extracts, making direct comparison among studies difficult [53,54,55].

Polar solvents such as methanol and ethanol generally produce extracts enriched in phenolic acids, flavonoids, and other polar secondary metabolites that are commonly associated with antioxidant and anti-inflammatory activities [56]. In contrast, non-polar solvents such as hexane preferentially extract fatty acids and other lipophilic constituents, particularly ricinoleic acid and related lipids, which are important for pharmaceutical formulations and industrial applications. Hydrodistillation selectively isolates volatile terpenoids, whereas modern techniques including ultrasound-assisted extraction, microwave-assisted extraction, and supercritical fluid extraction frequently improve extraction efficiency, reduce solvent consumption, and enhance recovery of thermolabile compounds [57].

Despite these technological advances, there remains no universally accepted extraction protocol for *R. communis*. Consequently, extracts described using the same solvent may differ substantially in chemical composition because of variation in plant material, geographical origin, cultivar, harvesting season, drying conditions, extraction parameters, and analytical methods. This methodological heterogeneity represents one of the major sources of conflicting pharmacological evidence identified throughout this review.

Table 2 summarizes the principal phytochemicals identified in different parts of *R. communis*, together with their extraction methods, analytical techniques, and reported biological significance. While this information provides a valuable overview of the phytochemical diversity of the species, it also illustrates an important limitation of the existing literature. Most studies identify compounds using chromatographic techniques but do not quantify their abundance sufficiently to establish reliable relationships between concentration and biological activity. Furthermore, relatively few investigations compare multiple extraction methods under standardized conditions or evaluate batch-to-batch reproducibility.

## 5. Toxicity of *R. communis* L.

*R. communis* presents several notable disadvantages that limit its safe cultivation and use despite its economic value.

### 5.1. Toxic Constituents and Toxicodynamics

Despite its extensive medicinal, industrial, and agricultural applications, *R. communis* presents significant toxicological concerns that require careful consideration. Toxicity is primarily associated with the seeds, which contain ricin, ricinine and *Ricinus communis* agglutinin 120 (RCA_120_), whereas other plant parts such as leaves, roots, and processed oils generally exhibit substantially lower toxicity levels. Ricin is a highly potent type II ribosome-inactivating protein composed of two polypeptide chains linked by a disulfide bond. The B-chain facilitates cellular attachment and internalization through binding to cell surface glycoproteins, while the A-chain irreversibly inhibits protein synthesis by depurinating a specific adenine residue within the 28S ribosomal RNA. This disruption of protein synthesis ultimately leads to apoptosis, tissue necrosis, organ dysfunction, and, in severe cases, death. The toxic effects of ricin are dose-dependent and vary according to the route of exposure, including ingestion, inhalation, and injection [72,73].

### 5.2. Routes of Exposure and Clinical Manifestations

Raw castor seeds constitute the greatest toxicological risk because ricin is concentrated mainly within the seed endosperm. Human poisoning most commonly occurs through accidental or intentional ingestion of seeds. The severity of poisoning depends on the number of seeds consumed, the degree of mastication, individual susceptibility, and the quantity of toxin released during digestion. Although ingestion of one or two seeds may result in mild symptoms in adults, chewing several seeds can produce severe intoxication, particularly in children. Inhalation or injection of purified ricin poses an even greater risk because these routes allow rapid systemic absorption. Experimental studies have estimated that the lethal dose of purified ricin may be as low as 5–10 μg/kg body weight, making it one of the most potent plant-derived toxins known. Clinical manifestations of ricin poisoning vary according to the route and extent of exposure. Oral exposure commonly produces severe abdominal pain, nausea, vomiting, diarrhea, and gastrointestinal hemorrhage. Progressive systemic toxicity may result in dehydration, electrolyte imbalance, hepatic injury, nephrotoxicity, hypotension, respiratory distress, and circulatory collapse. Human case reports indicate that the clinical outcome depends largely on the absorbed dose, degree of seed mastication, and the promptness of medical intervention [74,75,76].

### 5.3. Toxicity of Different Plant Parts and Products

The toxicological profile of *R. communis* varies considerably among plant-derived products. Properly processed castor oil is generally regarded as safe because ricin is water-soluble and remains in the seed cake during extraction rather than partitioning into the oil fraction [77]. Consequently, pharmaceutical- and industrial-grade castor oil contains negligible or undetectable levels of ricin when produced under appropriate manufacturing conditions. In contrast, castor seed cake, a by-product of oil extraction, may retain substantial quantities of ricin and therefore poses a significant toxicological hazard if not adequately treated before use as animal feed or fertilizer [78].

Although leaf and root extracts are frequently employed in traditional medicine and have demonstrated antioxidant, anti-inflammatory, antimicrobial, and wound-healing activities, their safety cannot be assumed. The phytochemical composition of these extracts varies according to plant part, extraction solvent, geographical origin, and environmental conditions [79]. The detection of potentially toxic constituents such as α-thujone in some *R. communis* extracts suggests that toxicity assessment remains essential before therapeutic application, emphasizing that natural products are often incorrectly perceived as inherently safe and demonstrating, through in silico toxicological screening, that certain phytochemicals isolated from *R. communis* may possess adverse toxicological properties [80]. These findings highlight the need for comprehensive biosafety evaluations before clinical development of *R. communis*-derived products.

### 5.4. Detoxification and Risk Mitigation Strategies

Given the economic importance of *R. communis*, considerable effort has been devoted to developing detoxification strategies for ricin-containing by-products. Several approaches have been investigated, including thermal treatment, autoclaving, alkaline hydrolysis, microbial fermentation, enzymatic degradation, and chemical processing, all of which aim to reduce ricin content while preserving the nutritional and agronomic value of castor seed cake. These detoxification methods are particularly important for the safe utilization of castor by-products in livestock feeding and agricultural applications. In addition, occupational safety measures, personal protective equipment, controlled handling procedures, and strict quality-control regulations are essential to minimize exposure risks during cultivation, processing, and industrial utilization of *R. communis* products [81,82].

### 5.5. Cytotoxicity and Safety Evaluation

The cytotoxic potential of *R. communis* extracts has been evaluated using the 3-(4,5-dimethylthiazol-2-yl)-2,5-diphenyltetrazolium bromide (MTT) cell proliferation assay across different cell lines (Table 3). Chloroform and ethyl acetate extracts demonstrated moderate cytotoxic activity against A549 lung carcinoma cells, suggesting that bioactive compounds present in the plant may possess antiproliferative properties. However, cytotoxic responses varied considerably depending on the extraction method, phytochemical composition, concentration, and cell type examined. While these findings support the potential development of *R. communis*-derived anticancer agents, they also underscore the need for careful dose optimization and safety assessment to distinguish therapeutic activity from nonspecific toxicity.

### 5.6. Clinical Management and Regulatory Considerations

Currently, no specific antidote for ricin poisoning has received regulatory approval, and treatment remains largely supportive. Clinical management focuses on early decontamination, maintenance of airway and circulatory function, correction of fluid and electrolyte disturbances, respiratory support, hemodynamic stabilization, and management of multiple organ dysfunction. Activated charcoal may reduce toxin absorption following recent ingestion, whereas severe poisoning frequently requires intensive care management. Clinical management focuses on early decontamination, fluid and electrolyte replacement, respiratory support, haemodynamic stabilization, and management of organ dysfunction. Activated charcoal may be beneficial following recent ingestion, while intensive care monitoring is often required in severe poisoning cases [86]. Given the growing medicinal and industrial interest in *R. communis*, adherence to biosafety regulations, proper processing procedures, occupational exposure controls, and rigorous quality assurance measures are essential to ensure safe utilization [87]. Therefore, although *R. communis* possesses considerable therapeutic and industrial potential, its safe application requires a clear distinction between toxic and non-toxic plant fractions, effective detoxification technologies, and comprehensive toxicological evaluation before pharmaceutical, nutraceutical, or industrial use.

### 5.7. Dose-Dependent Toxicity and Therapeutic Window

An important limitation of the current literature is the tendency to describe *R. communis* as either medicinal or toxic without adequately considering dose–response relationships. Like many pharmacologically active natural products, biological effects are highly dose-dependent. Low concentrations of specific phytochemicals may produce beneficial pharmacological responses, whereas higher concentrations or poorly purified preparations may result in cytotoxicity or systemic toxicity.

Unfortunately, relatively few studies have established therapeutic indices, no-observed-adverse-effect levels (NOAELs), or maximum tolerated doses for individual *R. communis* extracts. Moreover, differences in extraction procedures, phytochemical composition, experimental models, and dosing regimens complicate comparisons among investigations. Future studies should therefore prioritize standardized dose–response evaluations to distinguish therapeutic activity from nonspecific toxicity and to facilitate future clinical translation.

## 6. Traditional Uses of *R. communis* L.

**Figure 2:**
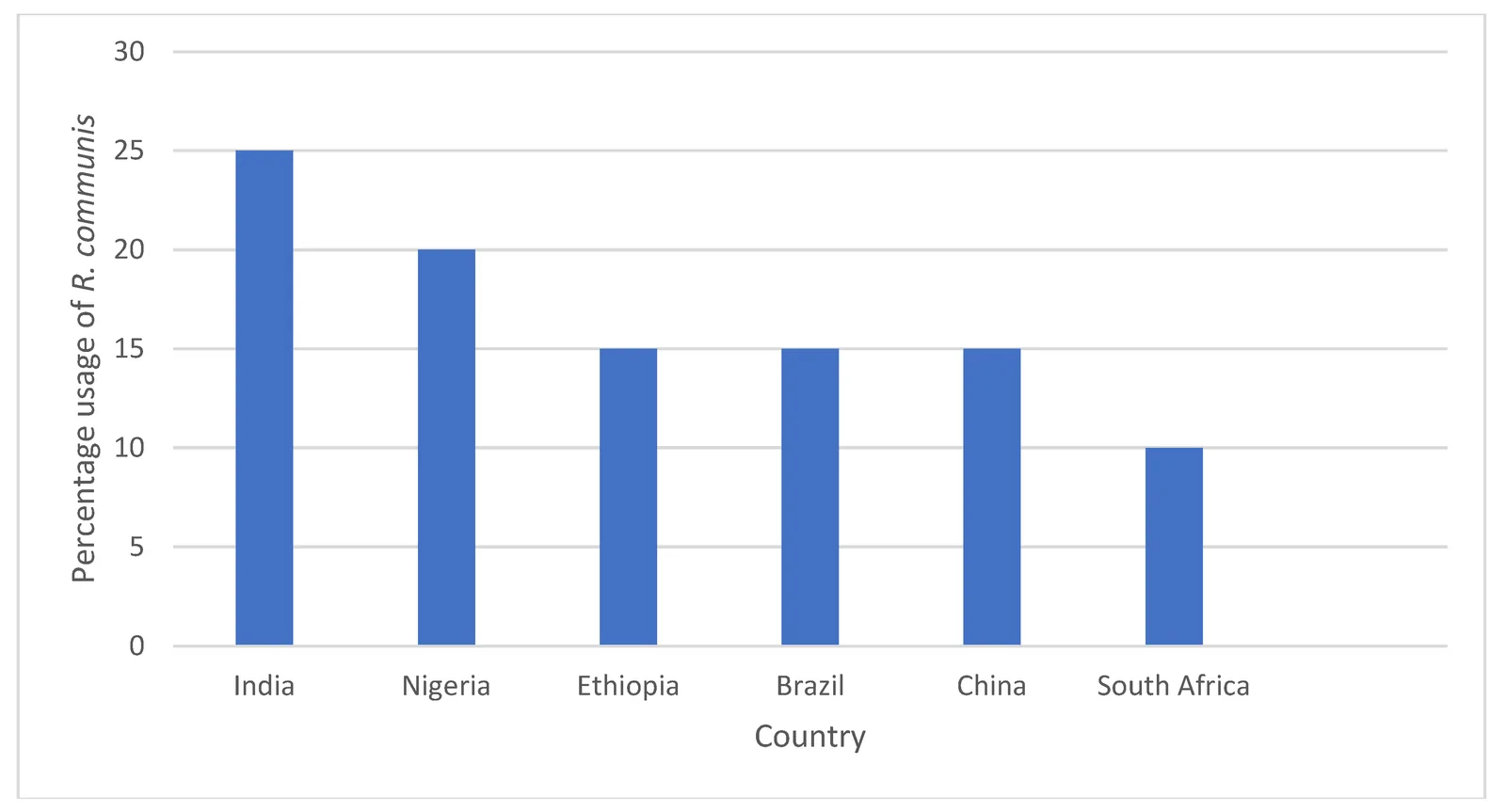
Geographic distribution of the documented traditional medicinal use of *R. communis* across selected countries. The percentages shown are based on the relative frequency of ethnomedicinal reports identified from the literature included in this review and are intended to illustrate the comparative extent of documented traditional use rather than absolute prevalence within each country. The figure highlights regions where *R. communis* has been most frequently reported in traditional healthcare practices.

*R. communis* has been used in traditional medicine for centuries and remains one of the most versatile medicinal plants employed across Africa, Asia, and South America (Figure 2). Virtually every part of the plant, including the leaves, roots, bark, seeds, and castor oil, has been incorporated into indigenous healthcare systems for the treatment of a wide range of ailments [88]. Although many traditional applications have subsequently attracted pharmacological investigation, the level of scientific evidence supporting these uses varies considerably. Some ethnomedicinal claims have been validated through experimental studies, whereas others remain supported primarily by traditional knowledge.

Traditional healers have employed the leaves as poultices or decoctions to treat skin sores, boils, and swellings, and have applied warmed, oil-coated leaves to the abdomen to relieve flatulence and stomach-ache [89]. In some African cultures, the leaves are also used to boost milk production in nursing mothers [90].

The roots are traditionally boiled or crushed into a paste for treating lumbago, sciatica, toothache, and other deep-seated pains [91]. These applications are broadly consistent with experimental studies reporting analgesic and anti-inflammatory activities of root extracts. Nevertheless, the active constituents responsible for these effects have not been fully characterized, and relatively few investigations have evaluated the efficacy or safety of traditional root preparations in humans.

Meanwhile, the bark is often crushed and applied to wounds to promote healing, showing its value in topically treating skin injuries. In addition to external applications, the seeds, once processed into castor oil, are used as a potent laxative to relieve constipation, and in some systems, they are administered in a controlled manner (after oil extraction) to stimulate digestion [92].

In addition, traditional medicine records include the use of castor oil for inducing labor, expelling placental matter, and as a purgative in children and the elderly [93]. While the laxative effect of ricinoleic acid has been extensively investigated and is supported by substantial pharmacological evidence, obstetric applications remain controversial because of inconsistent clinical outcomes and potential maternal and fetal risks. Consequently, the use of castor oil for labor induction should be approached cautiously and only under appropriate medical supervision.

Topical applications constitute another important component of traditional medicine. Castor oil is frequently applied to burns, wounds, eczema, dermatitis, acne, and other dermatological conditions because of its emollient, moisturizing, and antimicrobial properties [94,95]. In many African traditional healthcare systems, the oil is also massaged into painful joints and muscles to alleviate rheumatic pain and arthritis [96]. Experimental studies demonstrated that anti-inflammatory and wound-healing properties provide biological plausibility for these traditional practices; however, controlled clinical trials confirming their efficacy remain limited.

Some traditional uses have received comparatively little scientific attention. Fresh leaf juice has been administered as an emetic following poisoning or overdose in certain indigenous healthcare systems, while infusions have also been incorporated into ritual cleansing ceremonies in several cultures. These practices remain poorly investigated scientifically, and there is currently insufficient evidence to establish either their efficacy or safety. Furthermore, because phytochemical composition varies according to plant part, geographical origin, extraction method, and cultivar, the biological effects of traditional preparations may differ substantially from those reported in controlled laboratory studies.

Overall, the ethnomedicinal history of *R. communis* has provided an important foundation for modern pharmacological research and continues to guide the discovery of biologically active compounds. Nevertheless, the strength of evidence supporting individual traditional uses is highly variable. Applications relating to constipation, inflammation, wound healing, and pain management are supported by moderate preclinical evidence, whereas many other traditional claims remain.

## 7. Pharmacological Properties of *R. communis* L.

*R. communis* is a medicinal species that has attracted significant scientific attention due to its wide range of pharmacological properties. These biological activities are largely attributed to its diverse phytochemical composition, which include flavonoids, alkaloids, saponins, and phenolic compounds found in different parts of the plant, such as the seeds, leaves, and roots, using different extract types. Over the years, numerous in vitro and in vivo studies have validated its therapeutic potential, demonstrating activities relevant to the management of both acute and chronic diseases. The plant exhibits multiple pharmacological effects, including antioxidant, anti-inflammatory, antimicrobial, wound healing, anticancer, hepatoprotective, and antidiabetic activities, which collectively highlight its importance as a source of bioactive compounds for drug development and traditional medicine applications [97]. However, the strength of evidence supporting these activities varies considerably. Most available studies remain limited to preliminary in vitro investigations or small animal experiments, while robust clinical evidence is scarce. Furthermore, variations in extraction methods, phytochemical composition, dosage regimens, experimental models, and outcome measures make direct comparisons among studies difficult. Consequently, although *R. communis* exhibits promising pharmacological potential, the translational relevance of many reported bioactivities remains uncertain and requires further validation through standardized preclinical and clinical investigations.

### 7.1. Antioxidant Activity

Oxidative stress contributes significantly to the pathogenesis of chronic diseases including cancer, diabetes mellitus, cardiovascular disease, neurodegenerative disorders, and chronic inflammatory conditions. Consequently, natural antioxidants capable of scavenging reactive oxygen species and enhancing endogenous antioxidant defenses have attracted considerable pharmaceutical interest.

The antioxidant activity of *R. communis* is primarily attributed to phenolic acids, flavonoids, tannins, and other polyphenolic constituents present predominantly in the leaves. These compounds neutralize reactive oxygen species through electron donation, hydrogen atom transfer, metal ion chelation, and modulation of endogenous antioxidant defense systems [98,99]. Experimental evidence also suggests activation of the Nuclear factor erythroid 2-related factor 2/antioxidant response element (Nrf2/ARE) signaling pathway, resulting in increased expression of antioxidant enzymes including superoxide dismutase (SOD), catalase (CAT), and glutathione peroxidase (GPx), although confirmation in human studies remains unavailable.

Most evidence has been generated using in vitro antioxidant assays such as 2,2-Diphenyl-1-picrylhydrazyl (DPPH), 2,2′-Azino-bis(3-ethylbenzothiazoline-6-sulfonic acid) (ABTS), Ferric Reducing Antioxidant Power (FRAP), nitric oxide scavenging, and lipid peroxidation inhibition. Methanolic leaf extracts consistently demonstrate greater antioxidant capacity than seed extracts, largely because of their higher phenolic and flavonoid contents. For example, methanolic leaf extracts exhibited total phenolic contents of 189.5 ± 4.2 mg GAE/g, total flavonoid contents of 68.3 ± 2.1 mg QE/g, DPPH IC_50_ values of 0.14 ± 0.02 mg/mL, and inhibition of linoleic acid oxidation exceeding 82%. Soxhlet methanolic extraction further improved antioxidant activity, producing DPPH IC_50_ values of 0.121 ± 0.02 mg/mL. By comparison, ethanolic seed extracts generally showed lower antioxidant potency, with DPPH IC_50_ values of approximately 0.28 ± 0.05 mg/mL [100,101].

Although these findings consistently demonstrate substantial antioxidant activity, several limitations reduce confidence in the available evidence. Most studies rely exclusively on chemical antioxidant assays that do not necessarily predict biological efficacy in living systems. Experimental protocols, extraction methods, solvent selection, and phytochemical composition vary considerably between studies, making direct comparisons difficult. Furthermore, relatively few investigations correlate antioxidant activity with quantitative phytochemical profiles or validate the findings using animal models, and no controlled human clinical trials have been reported. Therefore, while antioxidant activity represents one of the best-documented biological properties of *R. communis*, its clinical significance remains uncertain.

### 7.2. Anti-Inflammatory Activity

Evidence from animal models is encouraging. The anti-inflammatory activity of *R. communis* has been demonstrated mainly in animal models, with leaf and root extracts reducing edema, granuloma formation, and inflammatory mediator release. In carrageenan-induced paw edema, hydroalcoholic leaf extract at 200 mg/kg reduced paw edema by 58% after 4 h, compared with a 62% reduction for indomethacin, the positive control, which suggests meaningful acute anti-inflammatory potential. In a cotton pellet granuloma model, root extract at 100 mg/kg reduced granuloma weight by 45%, indicating suppression of chronic proliferative inflammation. Other studies also used 250 and 500 mg/kg doses of methanolic leaf extract, with flavonoid fractions tested at 25, 50, and 100 mg/kg; these studies typically included normal saline as a negative control and indomethacin as a positive control. The proposed mechanisms include cyclooxygenase (COX) and lipoxygenase (LOX) inhibition, reduced nitric oxide (NO) production, and suppression of cytokines such as tumor necrosis factor alpha (TNF-α), interleukin-6 (IL-6), interleukin-1 beta (IL-1β), and interleukin-17A (IL-17A)., but the evidence remains preclinical and is limited by small sample sizes and variable study quality [102,103].

Despite these promising findings, interpretation should remain cautious. Most studies involve relatively small sample sizes, inconsistent experimental protocols, limited mechanistic validation, and short treatment durations. Dose justification, randomization, blinding, and reproducibility are seldom reported. Furthermore, no high-quality clinical trials have confirmed the anti-inflammatory efficacy of *R. communis* in humans. Consequently, current evidence supports moderate preclinical anti-inflammatory potential but remains insufficient for clinical recommendation.

### 7.3. Antimicrobial Activity

The emergence of antimicrobial resistance has intensified the search for plant-derived antimicrobial agents capable of complementing or enhancing existing therapeutic strategies. *R. communis* has demonstrated broad-spectrum antimicrobial activity against several clinically important bacterial and fungal pathogens, although the reported efficacy varies considerably depending on the plant part, extraction solvent, phytochemical composition, and microbial strain evaluated [104]. The antimicrobial activity of *R. communis* is primarily attributed to flavonoids, alkaloids, phenolic compounds, tannins, saponins, terpenoids, and ricinine, which act through multiple mechanisms. Proposed mechanisms include disruption of microbial cell membranes, alteration of membrane permeability, inhibition of essential microbial enzymes, interference with nucleic acid synthesis, and induction of oxidative stress within microbial cells [105].

Among the different plant parts investigated, the methanolic leaf extract showed the strongest activity against *Staphylococcus aureus*, with a zone of inhibition of 22 ± 0.2 mm and minimum inhibitory concentration (MIC) of 12.5 mg/mL, while *Escherichia coli* showed a zone of 19 ± 0.3 mm and MIC of 25.0 mg/mL, and *P. aeruginosa* was least sensitive with a zone of 9 ± 0.3 mm and MIC of 50.0 mg/mL. These studies generally used standard culture conditions but often lacked sufficient detail on reference antimicrobials, inoculum standardization, or solvent controls, which makes cross-study comparison difficult. The antimicrobial effect is usually linked to alkaloids, flavonoids, tannins, saponins, and phenolic compounds that may disrupt membranes and enzyme systems, but the evidence is still largely preliminary [106,107].

Despite these encouraging findings, antimicrobial evidence should be interpreted cautiously. Most studies remain confined to in vitro susceptibility assays, while relatively few investigate bactericidal mechanisms, biofilm inhibition, resistance modulation, or synergistic interactions with conventional antibiotics. Furthermore, methodological differences, including inoculum standardization, solvent selection, extract preparation, incubation conditions, and the use of positive controls, complicate comparisons among investigations. Most importantly, the concentration required to inhibit microbial growth is frequently higher than those of conventional antimicrobial agents, raising questions regarding therapeutic applicability.

Future investigations should prioritize bioactivity-guided isolation of antimicrobial compounds, mechanistic studies targeting resistant pathogens, evaluation against microbial biofilms, pharmacokinetic investigations, and controlled animal infection models. Clinical validation will ultimately be required before *R. communis*-derived antimicrobials can be considered for therapeutic applications.

### 7.4. Wound Healing Activity

Wound healing is a complex biological process involving coordinated inflammatory, proliferative, and remodeling phases that require appropriate regulation of oxidative stress, inflammation, angiogenesis, extracellular matrix deposition, and tissue regeneration. Traditional use of *R. communis* for wound management has stimulated numerous experimental investigations aimed at validating these therapeutic claims.

The wound-healing activity of *R. communis* is attributed primarily to ricinoleic acid, flavonoids, phenolic compounds, and triterpenoids, which collectively promote tissue repair through multiple complementary mechanisms. These include suppression of excessive inflammation, stimulation of fibroblast proliferation, enhanced collagen synthesis, increased angiogenesis, acceleration of epithelialization, and maintenance of a moist wound environment favorable for tissue regeneration [108]. In addition, the antimicrobial properties of the plant may reduce microbial colonization and thereby facilitate wound repair.

Experimental evidence supports these mechanisms. In a full-thickness rabbit wound model with 16 animals, topical leaf extract improved wound contraction to about 85% by day 14 compared with 70% in untreated controls, although the difference was not statistically significant. Histology showed reduced inflammation by day 3, better angiogenesis and collagen organization by day 7, and pronounced vascularization by day 14, which suggests support of the proliferative and remodeling phases of healing. In a rat excision wound model, castor oil produced 92% wound contraction by day 10 versus 78% in controls and increased tensile strength by 35%, supporting a stronger effect on tissue repair. These findings are promising, but the available studies are mostly small and do not always report blinding, power calculations, or robust statistical comparisons [109,110].

Although these findings provide biological support for traditional wound-healing applications, several limitations reduce confidence in the available evidence. Most studies involve relatively small numbers of experimental animals, short observation periods, and inconsistent reporting of randomization, blinding, statistical power, and histological assessment. Furthermore, differences in wound models, treatment protocols, and outcome measures make direct comparisons difficult. Importantly, robust clinical trials evaluating *R. communis*-based wound therapies in humans remain largely absent.

Future research should focus on standardized topical formulations, quantitative histological evaluation, molecular investigations of tissue repair pathways, chronic wound models, and well-designed clinical trials to establish therapeutic efficacy and safety in human patients.

### 7.5. Anticancer Activity

Among the pharmacological properties of *R. communis*, anticancer activity has attracted considerable scientific interest because of the potent biological effects of ricin and several non-protein phytochemicals. However, this area also requires the greatest caution because the therapeutic potential of the plant is closely associated with its toxicological profile.

The principal anticancer mechanism involves ricin, a type II ribosome-inactivating protein that irreversibly depurates 28S ribosomal RNA, thereby inhibiting protein synthesis and inducing apoptosis [111]. Additional mechanisms proposed for crude extracts include activation of caspase-dependent apoptosis, disruption of mitochondrial membrane potential, cell-cycle arrest, induction of oxidative stress, inhibition of tumor cell migration, modulation of autophagy, and suppression of proliferative signaling pathways. Flavonoids and phenolic compounds may further contribute through antioxidant and anti-inflammatory mechanisms that indirectly influence tumor progression.

Experimental evidence demonstrates cytotoxic activity against several human cancer cell lines. In the A375 melanoma cell line, aqueous extract showed an half-maximal inhibitory concentration (IC_50_) of 48 µg/mL after 48 h, indicating moderate cytotoxicity. In MCF-7 breast cancer cells, methanolic seed extract showed IC_50_ values of 85 ± 4 µg/mL at 24 h and 62 ± 3 µg/mL at 48 h, demonstrating time-dependent cytotoxicity and increased apoptosis. In A549 lung cancer cells, crude ricin produced an IC_50_ of 53.65 ± 0.78 µg/mL after 48 h and was reported to induce apoptosis, reduce migration, and increase autophagy. Ricin immunotoxins were far more potent, with IC_50_ values of 0.5–2 ng/mL, but these are engineered targeted constructs rather than crude plant extracts. Because ricin is highly poisonous, the anticancer evidence should be framed as experimental only, with clear separation between crude extract cytotoxicity and targeted immunotoxin research [112,113,114,115].

Despite these promising observations, the available evidence requires careful interpretation. Most studies remain confined to cultured cancer cell lines, and relatively few have progressed to animal tumor models or clinical evaluation. Moreover, many published reports fail to distinguish between crude plant extracts, purified ricin, and engineered immunotoxins, despite their fundamentally different pharmacological and toxicological properties. Ricin immunotoxins represent highly specialized targeted therapeutics in which the toxin is chemically linked to tumor-specific antibodies to minimize systemic toxicity. Their biological behavior therefore cannot be extrapolated directly to crude *R. communis* extracts.

Another important limitation is the narrow therapeutic window of ricin itself. Although highly effective at killing malignant cells, ricin is equally capable of damaging healthy tissues if delivered non-selectively. Consequently, current anticancer research has shifted towards targeted delivery systems, antibody-drug conjugates, nanoparticle-based formulations, and recombinant immunotoxins designed to maximize tumor selectivity while reducing systemic toxicity.

### 7.6. Hepatoprotective Activity

Liver diseases remain a major global health concern, with oxidative stress, chronic inflammation, and xenobiotic-induced hepatotoxicity contributing significantly to hepatic injury. Natural products possessing antioxidant and anti-inflammatory properties have therefore attracted considerable attention as potential hepatoprotective agents. Experimental evidence indicates that *R. communis* exhibits hepatoprotective activity, primarily through its ability to reduce oxidative damage, suppress inflammatory responses, and preserve hepatocellular integrity [116].

The hepatoprotective effects of *R. communis* are mainly attributed to phenolic compounds, flavonoids, and other antioxidant constituents present in the leaves and roots. These phytochemicals reduce lipid peroxidation, enhance endogenous antioxidant enzyme activity, preserve glutathione homeostasis, and suppress inflammatory mediators involved in hepatic injury. Experimental studies also suggest modulation of the Nrf2 signaling pathway and inhibition of NF-κB activation, thereby reducing oxidative stress and inflammatory damage within hepatic tissues.

For example, in one aqueous extract study, rats were divided into six groups: normal saline control, CCl_4_ control, Liv-52 positive control at 100 mg/kg, and test groups receiving *R. communis* at 250 and 500 mg/kg orally for 20 days; CCl_4_ was administered on days 2, 5, and 8. Both extract doses reduced Alanine aminotransferase (ALT) and aspartate aminotransferase (AST) significantly, with stronger effects at 500 mg/kg. In another rat model, methanolic leaf extract at 200 and 400 mg/kg reduced ALT, AST, alkaline phosphatase (ALP), and malondialdehyde (MDA) levels while increasing superoxide dismutase (SOD) and reduced glutathione (GSH), with 400 mg/kg performing closer to silymarin. These studies suggest antioxidant and membrane-stabilizing mechanisms, but the evidence is still preclinical, and no human hepatoprotective trials were identified [100].

Despite these promising findings, the evidence remains limited to experimental animal models. Considerable variation exists among studies regarding extraction methods, treatment duration, dose selection, and experimental design. Furthermore, the active compounds responsible for hepatoprotection have not been fully characterized, and pharmacokinetic data remain scarce. No controlled clinical trials have evaluated the hepatoprotective efficacy of *R. communis* in humans. Consequently, while current evidence suggests moderate preclinical hepatoprotective potential, clinical translation remains premature.

Future investigations should combine metabolomic profiling with mechanistic studies to identify the phytochemicals responsible for hepatoprotection, establish dose–response relationships, evaluate long-term safety, and determine whether these experimental findings can be reproduced in human clinical studies.

### 7.7. Antidiabetic Activity

The global increase in diabetes mellitus has stimulated extensive research into medicinal plants capable of improving glycemic control through multiple complementary mechanisms. Traditional use of *R. communis* for managing diabetes has encouraged numerous pharmacological investigations, although the available evidence remains predominantly preclinical.

Current evidence suggests that *R. communis* exerts antidiabetic activity through several mechanisms. Flavonoids, phenolic compounds, alkaloids, and other antioxidant constituents may reduce oxidative stress associated with pancreatic β-cell dysfunction, improve insulin sensitivity, inhibit intestinal carbohydrate-digesting enzymes such as α-amylase and α-glucosidase, and modulate glucose uptake in peripheral tissues.

*R. communis* has demonstrated significant antidiabetic potential in both streptozotocin- and alloxan-induced diabetic rat models. In one study, ethanolic leaf extract at 600 mg/kg reduced blood glucose by about 32%, with effects comparable to glibenclamide, and improved liver and kidney biochemical markers. Other studies reported that ethanol root extract lowered fasting blood glucose from 379 ± 72 mg/dL to 149 ± 11 mg/dL after 20 days, while another showed a 61.97% reduction within seven days. The proposed mechanisms include insulin secretion, β-cell protection, and inhibition of glucose absorption, with flavonoids, alkaloids, saponins, rutin, and α-thujone implicated as active constituents. Because the studies are confined to animal models and do not consistently report dose justification or controls, the findings should be described as promising but preliminary [117,118].

Although these findings are encouraging, important limitations remain. Most investigations employ chemically induced diabetes models that do not fully reproduce the complex pathophysiology of human Type 2 diabetes mellitus. Sample sizes are generally small, treatment periods are relatively short, and few studies compare *R. communis* directly with standard antidiabetic drugs using equivalent dosing strategies. Moreover, the phytochemical constituents responsible for glucose-lowering effects have not been conclusively identified, and the influence of cultivar variation on antidiabetic activity remains largely unexplored.

At present, no robust clinical evidence supports the use of *R. communis* for diabetes management in humans. Future research should prioritize bioactivity-guided isolation of active compounds, molecular investigations of insulin signaling pathways, pharmacokinetic studies, chronic toxicity evaluation, and well-designed randomized clinical trials before therapeutic recommendations can be made.

### 7.8. Analgesic (Pain Receptor Modulation)

Pain management represents one of the oldest traditional applications of *R. communis*, particularly in African and Asian traditional medicine, where leaf, root, and castor oil preparations have long been used to alleviate musculoskeletal pain, rheumatism, arthritis, and inflammatory disorders [119].

The analgesic activity of *R. communis* is believed to result primarily from the anti-inflammatory actions of flavonoids, phenolic compounds, triterpenoids, and ricinoleic acid. Proposed mechanisms include inhibition of cyclooxygenase-mediated prostaglandin synthesis, suppression of inflammatory cytokines, modulation of nitric oxide production, and reduced activation of peripheral nociceptors. These mechanisms suggest that analgesic activity is closely linked to the plant’s anti-inflammatory properties rather than direct effects on central opioid pathways.

Methanol leaf extract was tested at 100, 125, and 150 mg/kg in mice, while other reports used hydroethanolic seed extract at 200 mg/kg and aqueous root bark extract at 150 mg/kg. Ricinoleic acid showed 35% inhibition in the early formalin phase and 62% inhibition in the late inflammatory phase at 100 mg/kg, suggesting stronger activity against inflammatory pain. The seed extract increased tail-flick latency by 58% at 200 mg/kg, and root bark extract increased hot-plate latency by 51% at 150 mg/kg. Some studies used naloxone as an antagonist to show opioid involvement, whereas others suggested non-opioid mechanisms such as nitric oxide inhibition, calcium-channel blockade, and transient Receptor Potential Vanilloid 1 (TRPV1)-related desensitization. The evidence supports preclinical analgesic potential, but direct target validation and human confirmation are still lacking [120,121,122].

Despite these promising observations, the available evidence remains limited by methodological heterogeneity. Differences in extraction procedures, animal species, dosing regimens, and pain models hinder direct comparison among studies. Furthermore, few investigations have identified the specific phytochemicals responsible for analgesic activity, and mechanistic studies remain relatively limited. Importantly, no adequately powered clinical trials have evaluated the analgesic efficacy of *R. communis* preparations in human patients.

Future research should clarify the molecular mechanisms underlying analgesia, identify the principal bioactive compounds, investigate potential synergistic interactions among phytochemicals, and evaluate standardized formulations in controlled clinical studies.

### 7.9. Evidence Quality Note

The pharmacological activities of *R. communis* reviewed are supported by varying levels of scientific evidence. Antioxidant, antimicrobial, and anticancer activities are derived predominantly from in vitro studies, providing important mechanistic insights but limited evidence of therapeutic efficacy in vivo. Anti-inflammatory, wound-healing, hepatoprotective, antidiabetic, and analgesic activities have been investigated mainly in animal models, which offer stronger preclinical support but remain subject to limitations associated with experimental design, dosing strategies, species differences, and the use of chemically induced disease models.

Overall, while the available evidence highlights the broad pharmacological potential of *R. communis*, most reported activities remain at the preclinical stage. Future research should prioritize standardized extract preparation, identification of active constituents through bioactivity-guided approaches, mechanistic validation, pharmacokinetic and toxicological studies, and well-designed randomized controlled clinical trials to facilitate the translation of promising laboratory findings into evidence-based therapeutic applications.

To provide a concise overview of the current state of evidence, Table 4 presents an evidence map summarizing the pharmacological activities of *R. communis* according to the highest level of experimental validation available (in vitro, in vivo, or human studies), together with the principal bioactive constituents, proposed mechanisms of action, and overall translational readiness. This evidence-based summary enables readers to rapidly identify well-supported therapeutic applications, recognize areas where evidence remains limited, and highlight priorities for future research and clinical development.

## 8. Industrial Applications of *R. communis* L.

**Figure 3:**
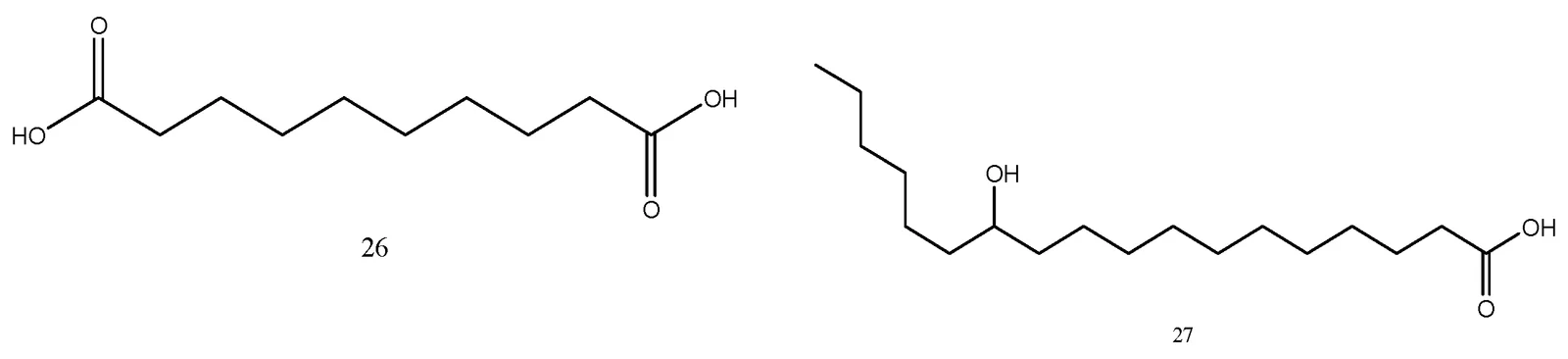
Specialty chemicals.

Industrially, the hydroxyl functionality of ricinoleic acid makes refined castor oil an exceptional renewable feedstock for producing specialty chemicals such as sebacic acid (26) and 12-hydroxystearic acid (27) (Figure 3) that are used in lubricants, plasticizers, surfactants and high-value polymer intermediates [123].

### 8.1. Processing Chain and Yields

Cultivation and seed supply are the foundational steps of the castor value chain; average seed yields vary substantially by region and agronomy but typically range from 0.5 to 2.0 t ha^−1^, and oil content commonly falls between 40 and 55% of seed weight. Seed handling and extraction choices determine both oil recovery and product quality: mechanical expeller pressing generally recovers 70–85% of the oil, whereas solvent extraction (hexane) can increase recovery to 90–98% at the cost of higher capital expenditure and solvent handling requirements; cold-pressing yields oil of higher cosmetic and pharmaceutical quality but with lower extraction efficiency. Refining and purification are required to meet pharmaceutical, cosmetic, or polymer feedstock specifications; degumming, neutralization, bleaching and deodorization raise processing costs and generate wastes such as soap stock and spent bleaching earth that must be treated or valorized. Downstream chemical conversions hydrolysis, transesterification for biodiesel, epoxidation, and selective transamination to produce monomers and oligomers exhibit variable yields: transesterification under optimized conditions typically exceeds 95% conversion, while selective modifications such as epoxidation report 70–95% yields depending on catalyst choice and operating conditions [124]. In pharmaceutical and cosmetic formulations, refined castor oil is commonly used as an excipient, emollient, and solubilizer due to its rheological properties and improved oxidative stability after refining [125,126,127].

### 8.2. Sustainability and Environmental Regulatory Considerations

From a sustainability perspective, the non-edible nature of castor reduces direct competition with food crops; however, land allocation, water use, agricultural inputs, and potential indirect land-use change remain important considerations that require region-specific life cycle assessment (LCA) studies. Although *R. communis* is widely regarded as a sustainable industrial feedstock, comprehensive environmental assessments across its pharmaceutical, cosmetic, and biofuel value chains remain limited, and its environmental benefits should therefore be interpreted within specific production contexts. The processing of castor seeds also presents significant safety and regulatory challenges due to the presence of ricin, a potent toxin, necessitating strict handling procedures, personal protective equipment, and, in some cases, detoxification processes that increase operational and compliance costs [128]. Waste streams generated during processing, including soap stock, spent bleaching earth, and glycerol, require effective management or valorization strategies to improve process sustainability, circularity, and economic feasibility. However, the relatively high viscosity and poor cold-flow properties of neat castor methyl esters often necessitate blending or further processing to achieve compliance with these standards [129]. Despite the considerable industrial potential of *R. communis*, additional studies integrating environmental sustainability, waste management, safety considerations, and regulatory compliance are needed to support its large-scale commercialization and long-term viability.

### 8.3. Polymers: Opportunities and Constraints

Castor oil is particularly valuable in polymer chemistry because the hydroxyl group of ricinoleic acid increases reactivity and allows direct synthesis of polyols, polyurethanes and specialty polyamides. Castor-derived monomers can produce materials with enhanced flexibility, improved biodegradability and reduced volatile organic compounds profiles [130]. Reports indicate castor-based polyurethanes often exhibit comparable elongation-at-break but lower glass transition metals (Tg) and somewhat reduced tensile strength relative to petrochemical analogs; crosslinking strategies and copolymerization are effective at mitigating these gaps. Economic constraints are important: the cost of refined castor oil and the reagents and catalysts for chemical modification frequently render castor-derived polymers more expensive at small scale. Industrial adoption therefore depends on securing reliable large-volume feedstock contracts, developing catalysts tolerant of impurities, and ensuring downstream processing is compatible with existing polymer manufacturing infrastructure [131,132].

### 8.4. Lubricants and Hydraulic Fluids

Castor derivatives, particularly ricinoleate esters, are valued for their high viscosity index, lubricity and biodegradability, and for maintaining more stable viscosity across temperature ranges than many vegetable oils. However, native castor oil exhibits poor low-temperature properties and may contain high acidity or impurities; chemical esterification and blending with synthetic esters or solvents are common routes to improve cold performance and stability, which increases formulation cost. Industrial deployment requires conformity with original equipment manufacturers specifications, compatibility testing with seals and paints, and long-term field tests to establish oxidation and hydrolytic stability under service conditions [133,134,135].

### 8.5. Pharmaceuticals and Cosmetics

*R. communis* is an important industrial crop with extensive applications in the pharmaceutical and cosmetic sectors, primarily due to its ricinoleic acid-rich castor oil. In pharmaceuticals, castor oil is widely used as a laxative, excipient, emulsifier, and carrier in drug delivery systems, while its derivatives are employed in the formulation of ointments, creams, and controlled-release medications [136,137]. In the cosmetic industry, castor oil is commonly utilized in skincare, haircare, and personal-care formulations because of its emollient, moisturizing, and conditioning effects. It is frequently incorporated into lotions, creams, soaps, lip products, shampoos, and hair conditioners, where it enhances product stability, texture, and hydration [138,139]. These diverse applications highlight the growing commercial significance of *R. communis* as a renewable source of functional ingredients for pharmaceutical and cosmetic formulations.

### 8.6. Biodiesel and Fuels

Castor’s high oil content and ricinoleic acid composition give biodiesel high lubricity, which is beneficial for engine wear [140]. The biodiesel production from *R. communis* is also considered a promising renewable energy pathway because the plant is a non-edible oilseed crop with high oil content, making it suitable for biodiesel without directly competing with food resources. The high viscosity and low pour point of castor oil biodiesel make it a versatile alternative fuel; however, its relatively high density often requires blending with conventional diesel to optimize engine performance [141]. Conversion metrics show that transesterification can provide fatty acid methyl ester (FAME) yields greater than 95% under optimized conditions, yet castor FAME often exceeds the kinematic viscosity limits in EN 14214 if used neat; typical industrial solutions are blending (e.g., B20–B50), partial hydrogenation, cracking, or other glyceride upgrading to reduce viscosity and improve cold-flow [142]. Industrial-scale barriers include securing a stable seed supply, managing ricin safety during processing, ensuring winter operability through appropriate blending/modification, and validating engine compatibility through durability testing [143].

Table 5 summarizes some of the major industrial sectors in which *R. communis* is utilized, together with their specific applications and industrial significance.

## 9. Additional Applications of *R. communis* L.

### 9.1. Weed Control (Herbicidal/Allelopathic) Applications

*R. communis* demonstrates potent herbicidal and allelopathic properties for weed control, primarily through its leaf extracts rich in phenolics and terpenoids [151]. In vitro studies have revealed that aqueous leaf extracts of *R. communis* inhibit the seed germination of weeds such as *Bidens bipinnata* by up to 100% at 25 g L^−1^ concentrations. These extracts also reduce seedling hypocotyl and radicle lengths via disruption of cell division and membrane permeability [152]. Foliar applications in pot experiments induced chlorosis, necrosis, reduced plant height and chlorophyll levels in *B. bipinnata*, with symptoms graded as strong phytotoxicity (grade 5) at 37.5 g L^−1^. These effects were linked to interference in photosynthesis and membrane permeability [153].

### 9.2. Tick Control (Acaricidal) Activity

*R. communis* demonstrates notable tick control (acaricidal) activity through both repellent and toxic effects against different tick species. Its essential oil has been shown to moderately repel ticks such as *Ixodes ricinus*, reducing their host-seeking behavior, which indicates interference with sensory or behavioral mechanisms [154]. More pronounced effects have been observed with crude extracts, particularly leaf extracts, where strong dose- and time-dependent mortality against ticks has been reported. Methanolic extracts are especially effective, achieving complete (100%) tick mortality at higher concentrations within relatively short exposure periods. Aqueous and ethanolic extracts also produce high mortality rates, although their efficacy is slightly lower compared to methanolic extracts [155].

### 9.3. Phytoremediation of Soil

*R. communis* has emerged as a promising phytoremediation species for contaminated soils because of its rapid growth, extensive root system, tolerance to abiotic stress, and ability to accumulate or stabilize pollutants without major reductions in biomass productivity. Across the reviewed studies, its application spans remediation of organic pollutants, heavy metals, mine tailings, saline soils, and chemically assisted remediation systems, showing that the plant can function through both phytoextraction and phytostabilization mechanisms [156,157].

One of the major reasons *R. communis* is valuable in phytoremediation is its high adaptability to harsh soil conditions [158]. The species is fast-growing, produces substantial biomass, and develops a deep, widespread root architecture capable of penetrating contaminated substrates and accessing pollutants beyond the superficial soil layer. This extensive root system increases contact with contaminated soil, improving pollutant uptake and stabilization efficiency. Additionally, the plant tolerates drought, salinity, and metal stress, which makes it particularly useful in degraded and marginal environments where conventional crops may fail [159].

Besides heavy metals, *R. communis* also demonstrates effectiveness in the remediation of persistent organic pollutants (POPs). Castor bean was investigated in greenhouse experiments involving soils contaminated with organochlorine pesticides. The study showed that castor could survive and grow in polluted soils while promoting pollutant reduction. Its phytoremediation effect was linked not only to direct uptake but also to interactions within the rhizosphere, where root-associated microbial activity may facilitate degradation of contaminants. Thus, *R. communis* contributes to remediation through both plant-based absorption and indirect stimulation of biodegradation processes [160]. Chelating agents such as citric acid (CA), ethylenediamine disuccinic acid (EDDS), and nitrilotriacetic acid (NTA) can improve heavy metal bioavailability in soils, enhancing uptake by *R. communis*. Research suggests that these chelators increase rhizospheric metal mobility and improve accumulation of metals like lead (Pb) and cadmium (Cd) in castor tissues, thereby increasing remediation efficiency. This indicates that *R. communis* can be integrated into engineered phytoremediation systems [161].

## 10. Clinical Studies of *R. communis* L.

Although extensive preclinical research has demonstrated diverse pharmacological activities of *R. communis*, translation into clinical practice remains limited. Most published studies have been confined to in vitro experiments or animal models, while well-designed human clinical trials evaluating efficacy, safety, optimal dosage, pharmacokinetics, and long-term outcomes remain scarce. Consequently, most therapeutic claims associated with *R. communis* should currently be regarded as promising preclinical observations rather than evidence-based clinical interventions [162].

### 10.1. Clinical Applications of Pharmaceutical-Grade Castor Oil

Among all *R. communis*-derived products, pharmaceutical-grade castor oil has the strongest clinical evidence and represents the only preparation widely incorporated into modern medical practice. Following oral administration, ricinoleic acid is released through enzymatic hydrolysis within the small intestine, where it activates prostaglandin EP3 receptors, stimulates intestinal smooth muscle contraction, increases electrolyte and water secretion, and accelerates intestinal transit [163]. These mechanisms account for its well-established use as a stimulant laxative for the short-term treatment of constipation.

Several clinical investigations have demonstrated that castor oil effectively improves bowel evacuation and reduces symptoms associated with functional constipation, particularly in elderly patients and individuals requiring bowel preparation before selected medical procedures. However, prolonged or excessive use may produce abdominal cramping, diarrhea, dehydration, electrolyte imbalance, and dependence on stimulant laxatives. Consequently, most clinical guidelines recommend castor oil only for short-term management under appropriate medical supervision [137,164]. Although experimental evidence strongly supports antimicrobial and wound-healing activities, high-quality randomized controlled trials evaluating topical *R. communis*-based formulations remain limited. Additional clinical studies are therefore required before definitive therapeutic recommendations can be established.

### 10.2. Clinical Development of Ricin-Based Anticancer Therapeutics

Unlike crude plant extracts, purified ricin has primarily been investigated as a component of targeted anticancer therapies. Recombinant immunotoxins and antibody–ricin conjugates have been developed to selectively deliver ricin to tumor cells while reducing systemic toxicity. Early phase clinical trials evaluating ricin-based immunotoxins have demonstrated selective tumor cell killing in certain hematological malignancies and solid tumors [165]. However, widespread clinical application has been limited by immunogenicity, vascular leak syndrome, off-target toxicity, and challenges associated with targeted delivery.

Current research focuses on improving tumor specificity through recombinant protein engineering, antibody optimization, nanoparticle-based delivery systems, and ligand-directed targeting strategies. These approaches aim to enhance therapeutic efficacy while minimizing adverse effects associated with systemic ricin exposure.

### 10.3. Safety Considerations in Human Use

Clinical use of *R. communis*-derived products requires careful distinction between pharmaceutical-grade castor oil and crude plant materials. Properly refined castor oil contains negligible amounts of ricin because the toxin is removed during industrial processing and does not partition into the oil fraction. Consequently, pharmaceutical-grade castor oil has an established safety profile when administered appropriately [166].

Conversely, ingestion of crude seeds, unrefined extracts, or ricin-containing plant materials may result in severe poisoning characterized by gastrointestinal distress, dehydration, multi-organ dysfunction, and potentially fatal outcomes [167]. These toxicological considerations emphasize the importance of rigorous quality control, validated manufacturing procedures, and compliance with Good Manufacturing Practice (GMP) standards for all pharmaceutical products derived from *R. communis*.

### 10.4. Limitations of Current Clinical Evidence

Despite decades of pharmacological research, the clinical evidence supporting most therapeutic applications of *R. communis* remains limited. Most available studies involve relatively small patient populations, short follow-up periods, heterogeneous treatment protocols, and variable outcome measures. Furthermore, many investigations evaluate castor oil rather than other phytochemically characterized plant extracts, creating a substantial gap between preclinical pharmacological discoveries and clinical application.

Another major limitation is the absence of standardized formulations for leaf, root, or seed extracts. Variability in phytochemical composition resulting from differences in cultivar, geographical origin, extraction method, and processing further complicates interpretation of published findings. These limitations currently restrict the reproducibility and generalizability of clinical outcomes.

To provide a clearer overview of the current state of clinical research, Table 6 summarizes the available clinical studies involving *R. communis* and its derivatives, highlighting the therapeutic application investigated, the formulation evaluated, study design, principal clinical findings, safety outcomes, and overall level of evidence. The table illustrates that, despite extensive preclinical evidence supporting the pharmacological potential of *R. communis*, only a limited number of applications, most notably pharmaceutical-grade castor oil as a stimulant laxative, have been supported by moderate to high-quality clinical evidence. In contrast, many other therapeutic applications remain at an early stage of clinical development, underscoring the need for standardized formulations, larger randomized controlled trials, and comprehensive long-term safety evaluations before broader clinical adoption can be recommended.

## 11. Future Directions and Research Gaps for *R. communis* L.

Although *R. communis* L. has shown a broad range of pharmacological and industrial potential, several key research gaps must be addressed before its applications can be translated more confidently into practice. A major priority is omics-guided phytochemical discovery, including cultivar-level metabolomic and chemotaxonomic profiling, to determine how genetic background, geography, harvesting stage, and plant part influence bioactive composition. This should be paired with standardized extraction and characterization protocols so that results across studies become comparable and reproducible. Future work should also focus on isolating and validating the specific compounds responsible for the reported activities, particularly those linked to antioxidant, anti-inflammatory, antidiabetic, antimicrobial, and anticancer effects, using high-throughput screening, bioassay-guided fractionation, and structure–activity relationship analysis.

Another important priority is mechanistic pharmacology. Many current studies report bioactivity without adequately defining the molecular pathways involved, so future research should identify targets such as inflammatory mediators, oxidative stress pathways, apoptotic signaling, glucose-regulating pathways, and microbial resistance mechanisms. In parallel, studies should move beyond preliminary screening and include validated in vivo models, with proper controls, dose justification, randomization, blinding, and statistical rigor. Because toxicity remains a central concern, especially due to ricin and related toxic constituents, future investigations should include acute, sub-chronic, and chronic toxicity studies, dose–response evaluation, organ-specific safety profiling, and risk assessment to determine safe exposure ranges.

There is also a clear need for human safety and clinical validation. Most existing evidence is preclinical, and the therapeutic relevance of many findings remains uncertain without pharmacokinetic, pharmacodynamic, and clinical studies in humans. Controlled trials are needed to assess efficacy, tolerability, formulation safety, and real-world applicability. At the same time, translational formulation research should explore castor-based products, purified fractions, and nanocarriers to improve solubility, stability, targeted delivery, and toxicity mitigation. Finally, future work should address industrial scalability and quality control, including batch-to-batch standardization, Good Manufacturing Practice-aligned production, stability testing, and yield optimization, to support reproducible pharmaceutical and industrial development.

## 12. Materials and Methods

This review article was carried out by searching electronic databases, including Google Scholar, Elsevier, MDPI, Scopus, PubChem and ScienceDirect, to identify and retrieve relevant articles and research papers of *R. communis* L. published from 2015 to 2026. All chemical structures were drawn using ChemDraw Ultra^®^ 8.0 Software.

## 13. Conclusions

This review highlights *R. communis* L. as a plant of exceptional phytochemical diversity and multifunctional relevance, bridging traditional medicine, pharmacological research, and industrial biotechnology. The dominance of ricinoleic acid in castor oil, together with the presence of alkaloids, phenolics, flavonoids, and bioactive proteins, provides a clear biochemical basis for many ethnomedicinal practices and experimentally validated pharmacological effects, particularly anti-inflammatory, antimicrobial, antioxidant, wound-healing, and laxative activities. Although taxonomically monotypic, *R. communis* exhibits marked intraspecific variation, with cultivar- and tissue-dependent differences in chemical composition, oil yield, and bioactivity. These variations present opportunities for targeted selection of cultivars for medicinal, ornamental, or industrial purposes. Industrially, castor oil remains a strategically important renewable resource, enabling the production of high-value chemicals, polymers, pharmaceuticals, cosmetics, and biofuels. Nonetheless, the inherent toxicity of ricin present in raw seeds continues to pose significant safety and regulatory challenges, thereby reinforcing the need for refined products and controlled processing.

## Figures and Tables

**Table 1:** Phytochemical biosynthesis pathways of *R. communis*.

Pathway Class	Core Precursor Route	Key Enzymes/Genes	Major Metabolites	References
Fatty acid and TAG biosynthesis	Plastid fatty acid synthesis followed by ER triacylglycerol assembly	ACCase, ACP, KAS, KAR, SAD, GPAT, LPAAT, DGAT, PDAT, FAH12	Ricinoleic acid-rich TAGs	[35,36]
Phenolamide biosynthesis	Hydroxycinnamoyl-CoA conjugation to amines	RcHCT1, RcHCT2	N-feruloyl putrescine, N-caffeoyl putrescine, N-caffeoyl serotonin	[37]
Flavonoid biosynthesis	Phenylpropanoid-derived flavonoid route	Flavonoid biosynthetic enzymes and transferases	Rutin, quercetin derivatives, kaempferol derivatives	[38]
Alkaloid-related metabolism	Specialized nitrogen metabolism	Upstream ricinine-forming enzymes	Ricinine	[39,40]

**Table 2:** Major phytochemical constituents identified in different parts of *R. communis* L., their extraction methods, analytical characterization, and biological significance.

Plant Part	Major Compound(s)	Extraction Method/Solvent	Analytical Method	Biological Relevance	References
Seed oil	Ricinoleic acid	Cold pressing, Soxhlet extraction (hexane)	GC-MS, GC-FID	Anti-inflammatory, analgesic, biodiesel feedstock, polymer production	[58,59]
Seeds	Ricin	Protein extraction, ammonium sulphate precipitation	SDS-PAGE, HPLC, LC-MS	Cytotoxic, anticancer research, highly toxic	[60,61]
Leaves, seeds	Ricinine	Methanol, ethanol extraction	HPLC, LC-MS	Insecticidal, antimicrobial, antioxidant, toxicological marker	[62,63]
Seeds	Oleic acid	Hexane extraction	GC-MS	Cardioprotective, industrial applications	[64]
Seeds	Linoleic acid	Hexane extraction	GC-MS	Antioxidant, antimicrobial	[65]
Leaves	Quercetin, Kaempferol, Gallic acid	Methanol, ethanol extraction	HPLC-DAD, LC-MS, HPLC	Antioxidant, anti-inflammatory, antimicrobial	[66,67,68]
Leaves	α-Thujone	Essential oil hydrodistillation	GC-MS	Bioactivity marker; potential toxicity concern	[69,70]
Stem bark	Tannins	Aqueous and methanol extraction	Spectrophotometric assays	Antioxidant and antimicrobial activities	[71]

**Table 3:** Evaluation of *R. communis* cytotoxicity on different cell lines.

Plant Part/Form	Cell Line(s) Tested	IC_50_ Value	Key Findings	Reference
Crude ricin (seed protein)	A549 (lung cancer)	918 ± 2.05 µg/m	Induced apoptosis via caspase pathways; inhibited migration and autophagy	[83]
Seed extract	MCF-7, A2780, HT29 (breast cancer, ovarian cancer, colorectal cancer)	1.52, 3.04, 3.95 µg/mL	Strong cytotoxicity; highest activity against MCF-7; good selectivity toward cancer cells	[84]
Leaf/root-mediated Ag nanoparticles	RBCs (haemolysis assay)	<20 µg/mL (safe range)	Low cytotoxicity at lower concentrations; indicates good biocompatibility	[85]

**Table 4:** Evidence map summarizing the pharmacological activities of *R. communis* L., level of experimental evidence, principal bioactive constituents, proposed mechanisms of action, and translational readiness.

Pharmacological Activity	Principal Bioactive Constituents	Proposed Mechanism(s)	In Vitro Evidence	In Vivo Evidence	Human Evidence	Overall Translational Readiness	Key Research Gaps
Antioxidant	Flavonoids, phenolic acids, tannins	ROS scavenging, metal chelation, activation of antioxidant enzymes	✓✓✓	✓	✗	Moderate	Clinical validation; standardized extracts
Anti-inflammatory	Flavonoids, ricinoleic acid, triterpenoids	COX/LOX inhibition, NF-κB suppression, cytokine modulation	✓✓	✓✓	✗	Moderate	Human efficacy and dose optimization
Antimicrobial	Ricinine, flavonoids, phenolics, tannins	Membrane disruption, enzyme inhibition, oxidative stress	✓✓✓	✓	✗	Low–Moderate	Animal infection models; clinical studies
Wound healing	Ricinoleic acid, flavonoids, phenolics	Enhanced collagen synthesis, angiogenesis, epithelialization	✓	✓✓	△	Moderate	Randomized clinical trials
Anticancer	Ricin, flavonoids, phenolics	Apoptosis induction, ribosome inactivation, cell-cycle arrest	✓✓✓	✓	△ *	Experimental	Safer targeted delivery systems; clinical validation
Hepatoprotective	Phenolics, flavonoids	Antioxidant and anti-inflammatory hepatoprotection	✓	✓✓	✗	Low–Moderate	Pharmacokinetics and clinical studies
Antidiabetic	Flavonoids, alkaloids, phenolics	α-Amylase/α-glucosidase inhibition, insulin sensitization	✓	✓✓	✗	Low–Moderate	Active compound identification; clinical trials
Analgesic	Ricinoleic acid, flavonoids	Prostaglandin inhibition, anti-inflammatory activity	✓	✓✓	✗	Moderate	Mechanistic and clinical investigations
Laxative (castor oil)	Ricinoleic acid	EP3 receptor activation, increased intestinal motility	✓✓	✓✓	✓✓✓	High	Long-term safety and optimized formulations

Key: ✓✓✓ = extensive evidence; ✓✓ = moderate evidence; ✓ = limited evidence; △ = preliminary or indirect human evidence; ✗ = no clinical evidence. * Human evidence for anticancer activity relates primarily to investigational ricin-based immunotoxins rather than crude *R. communis* extracts.

**Table 5:** Industrial use of *R. communis* L. in different sectors.

Sector	Industrial Use	Description	References
Pharmaceutical Industry	Castor oil	Used as a laxative and in topical applications for anti-inflammatory properties.	[144]
Cosmetics and Personal Care	Emollients	Act as a moisturizer and skin conditioner in cosmetic products.	[145,146]
Food Industry	Food additives	Occasionally used as a food additive and in flavoring.	[147]
Agriculture	Pesticides and herbicides	Used to develop natural pesticides and herbicides for sustainable agriculture.	[148]
Manufacturing	Lubricants, plastic and resins	Utilized in producing high-performance lubricants due to its viscosity and stability and key ingredients in biodegradable plastics and synthetic resins.	[149]
Textile industry	Textile fishing agents	Used in textile processing as softeners and finishing agents.	[150]

**Table 6:** Summary of clinical studies involving *R. communis* and castor oil.

Clinical Application	Product Evaluated	Study Type	Main Findings	Safety Outcome	Level of Evidence
Constipation	Pharmaceutical-grade castor oil	Randomized clinical trials	Improved bowel evacuation and reduced constipation symptoms	Mild gastrointestinal adverse effects	High
Bowel preparation	Castor oil	Clinical studies	Effective bowel cleansing before procedures	Generally, well tolerated	Moderate
Labor induction	Castor oil	Clinical trials and meta-analyses	Mixed efficacy; inconsistent findings	Nausea and diarrhea common	Moderate
Wound healing	Topical castor oil formulations	Small clinical studies	Improved skin hydration and healing	Minimal adverse effects	Low
Dermatological disorders	Castor oil formulations	Observational studies	Improved skin barrier function	Well tolerated	Low
Cancer therapy	Ricin immunotoxins	Phase I/II trials	Selective tumor targeting but toxicity remains a concern	Immunogenicity and vascular leak syndrome	Experimental

## Data Availability

No new data were created or analyzed in this study. Data sharing is not applicable to this article.

## Abbreviations

The following abbreviations are used in this manuscript:

**ABTS**	2,2′-Azino-bis(3-ethylbenzothiazoline-6-sulfonic acid)
**ALP**	Alkaline Phosphatase
**ALT**	Alanine Aminotransferase
**AST**	Aspartate Aminotransferase
**As**	Arsenic
**B. bipinnata**	*Bidens bipinnata*
**Ca^2+^**	Calcium Ion
**CA**	Citric Acid
**Cd**	Cadmium
**CFA**	Complete Freund’s Adjuvant
**COX**	Cyclooxygenase
**COX-2**	Cyclooxygenase-2
**CRP**	C-Reactive Protein
**Cu**	Copper
**DNA**	Deoxyribonucleic Acid
**DPPH**	2,2-Diphenyl-1-picrylhydrazyl
**EDDS**	Ethylenediamine-N,N′-disuccinic Acid
**EDTA**	Ethylenediaminetetraacetic Acid
**ER+**	Estrogen Receptor-Positive
**Fe**	Iron
**GABAA**	Gamma-Aminobutyric Acid Type A Receptor
**GC-FID**	Gas Chromatography–Flame Ionization Detection
**GC-MS**	Gas Chromatography–Mass Spectrometry
**GSH**	Reduced Glutathione
**HPLC**	High-Performance Liquid Chromatography
**HPLC-DAD**	High-Performance Liquid Chromatography with Diode Array Detection
**IC_50_**	Half-Maximal Inhibitory Concentration
**IFN-γ**	Interferon Gamma
**IL-1β**	Interleukin-1 Beta
**IL-4**	Interleukin-4
**IL-6**	Interleukin-6
**IL-17A**	Interleukin-17A
**LC-MS**	Liquid Chromatography–Mass Spectrometry
**LOX**	Lipoxygenase
**LCA**	Life Cycle Assessment
**MCF-7**	Michigan Cancer Foundation-7 Breast Cancer Cell Line
**MDA**	Malondialdehyde *(or MDA-MB-231 where applicable—see below)*
**MDA-MB-231**	MD Anderson Metastatic Breast Cancer-231 Cell Line
**MERCL**	Methanolic Extract of *Ricinus communis* Leaves
**MIC**	Minimum Inhibitory Concentration
**Mn**	Manganese
**MTT**	3-(4,5-Dimethylthiazol-2-yl)-2,5-diphenyltetrazolium Bromide
**NF-κB**	Nuclear Factor Kappa B
**NMDA**	N-Methyl-D-Aspartate
**NO**	Nitric Oxide
**Nrf2**	Nuclear factor erythroid 2-related factor 2
**NSAID**	Non-Steroidal Anti-Inflammatory Drug
**NTA**	Nitrilotriacetic Acid
**Pb**	Lead
**PGE_2_**	Prostaglandin E_2_
**POPs**	Persistent Organic Pollutants
**QE**	Quercetin Equivalents
**RCA**	*Ricinus communis* Agglutinin
**RCA120**	*Ricinus communis* Agglutinin 120
**RF**	Rheumatoid Factor
**RIP2**	Type 2 Ribosome-Inactivating Protein
**ROS**	Reactive Oxygen Species
**RANKL**	Receptor Activator of Nuclear Factor Kappa-B Ligand
***R. communis***	*Ricinus communis*
**SDS-PAGE**	Sodium Dodecyl Sulfate–Polyacrylamide Gel Electrophoresis
**SOD**	Superoxide Dismutase
**STZ**	Streptozotocin
**TF**	Translocation Factor
**TFC**	Total Flavonoid Content
**TNF-α**	Tumor Necrosis Factor Alpha
**TPC**	Total Phenolic Content
**TRPV1**	Transient Receptor Potential Vanilloid 1
**Zn**	Zinc
